# ENA-78 Is a Novel Predictor of Wound Healing in Patients with Diabetic Foot Ulcers

**DOI:** 10.1155/2019/2695436

**Published:** 2019-01-15

**Authors:** Ju-yi Li, Zhong-jing Wang, Ai-ping Deng, Yu-ming Li

**Affiliations:** ^1^Department of Pharmacy, The Central Hospital of Wuhan, Tongji Medical College, Huazhong University of Science and Technology, Wuhan, 430021 Hubei, China; ^2^Department of Endocrinology, The Central Hospital of Wuhan, Tongji Medical College, Huazhong University of Science and Technology, Wuhan, 430021 Hubei, China; ^3^Department of Endocrinology, Union Hospital, Tongji Medical College, Huazhong University of Science and Technology, Wuhan, 430022 Hubei, China

## Abstract

**Objectives:**

Chronic foot ulceration is a severe complication of diabetes, driving morbidity and mortality. The aim of our study was to identify novel biomarkers of impaired wound healing in diabetic foot ulcers.

**Methods:**

109 patients with neuropathic diabetic foot ulcers and 30 burn victims otherwise healthy participated. Antibody-coated glass slide arrays were used to determine the levels of 80 human cytokines in pooled plasma or pooled wound exudate of diabetic foot ulcers with rapidly healing (RH, *n* = 12) and matched nonhealing (NH, *n* = 12) patients. Potential biomarkers were confirmed in an independent cohort by enzyme-linked immunosorbent assay (ELISA).

**Results:**

Protein array profiling identified 27 proteins or 15 proteins significantly altered in protein profiling of pooled plasma or pooled wound exudate of 12 RH patients compared with 12 matched NH patients, respectively. In an independent cohort, quantitative ELISA validation confirmed a decrease in MCP-2 and ENA-78 levels in NH patients versus RH patients or burn victims. After adjusting for the traditional risk factors (sex, age, body mass index, fasting plasma glucose, ulcer area, HbA1C, diabetes duration, hyperlipidemia, and antibiotic therapy), only wound exudate level of ENA-78 remained having a significant association with an increased odds ratio (OR) for wound healing by binary logistic regression analysis (*P* < 0.05).

**Conclusion:**

Decreased wound exudate ENA-78 was independently associated with wound healing of patients with diabetic foot. Exudate ENA-78 level is implicated as a novel predictor of wound healing in patients with diabetic foot ulcers.

## 1. Introduction

Diabetic foot is a severe chronic diabetic complication and has become a major public health problem that consists of neurological disorders and peripheral vascular diseases in the lower extremities [1, 2]. Diabetic foot ulcers (DFU) are associated with increased rate of disability and mortality which are leading cause of nontraumatic amputation in developed countries and are also associated with heavy medical burden [3, 4]. Wound healing is a complex process involving several tissues, cell types, and biological pathways; the risk factors consist of coagulation, formation, and regression of the granulation tissue, epithelial gap closure, angiogenesis, and inflammation, but the molecular mechanisms leading to impaired wound healing in diabetes are incompletely understood [5].

Indeed, several studies have shown the clinical relevance of cytokines in wound exudate or plasma of DFU, such as MMP-9, TIMP-1, S100A8, S100A9, and TGF-*β* in wound exudate, exudate MMP-1/TIMP-1 ratio, serum MMP-9/TIMP-1 ratio [1, 3, 6–8]. However, the development of these biomarkers from bench-to-bedside is a lengthy process [9]. So far, studies on the mechanisms of impaired wound healing in diabetes have failed to transfer preclinical findings into clinical-grade therapeutic strategies. Because of the difficulty in obtaining tissue samples, the studies on DFU are limited; however, plasma or wound exudate is much easier to be obtained in clinical practice. Clearly, early recognition of wound healing and more valuable biomarkers in plasma or exudate are urgently required as early predictor markers of wound healing for reducing the high number of amputations.

High-throughput antibody arrays are designed for screening large numbers of protein biomarker in cell culture media, tissues, or body fluids, which are used in various fields including diabetes, cancer, autoimmune diseases, and cardiovascular diseases [9–11]. So far, proteomics including antibody arrays has rarely been used to discover new aspects of the impaired wound healing in diabetes, and the biomarker or resulting pathways have never been validated. However, patients with diabetic foot were followed up to evaluate wound healing, which will likely provide more reliable data on the molecular mechanisms underlying the healing process.

In the present study, a discovery study was conducted in plasma or exudate samples of patients with diabetic foot (12 rapidly healing (RH) patients and 12 matched nonhealing (NH) patients) using an antibody array containing 80 potential biomarker proteins (including chemokines, inflammatory cytokines, and angiogenesis-related factors). Based on these screening tests, many potential biomarkers were identified; MCP-2 and ENA-78 were validated via ELISA in the validation study. Subsequently, after adjusting for traditional confounding risk factors, our result suggests that decreased ENA-78 level in wound exudate is an independent predictor of wound healing of patients with diabetic foot.

## 2. Materials and Methods

### 2.1. Subjects

This study was approved by the Ethics Committee of the Central Hospital of Wuhan and was conducted in accordance with the principles of the Declaration of Helsinki as revised in 2000. Participating subjects gave their written informed consent to participate in the study. Patients with diabetic foot aged 20–80 years admitted to the inpatient department were recruited from the Central Hospital of Wuhan from October 2015 to May 2017. Diabetic patients with neuropathic wounds which were graded on a 2 to 3 scale according to the Texas Grading System [12], ankle/brachial index (ABI) from 0.9 to 1.3, and on arterial plaque in lower extremity examined by using color Doppler ultrasound could be enrolled. Foot ulcers were classified as previously described [5], which were graded on a 2 to 3 scale could be enrolled. In addition, 30 burn victims otherwise healthy (second-degree to third-degree burns on the thigh) aged 10–70 years admitted to the outpatient department were recruited from October 2015 to May 2016. All the participants had a wound with an area larger than 0.5 cm^2^. Wound size was estimated by digital photography using ImageJ2x (NIH, Bethesda, MD, USA). Exclusion criteria were systemic infection or sepsis, cancer, immunological disorders, pregnancy, or lactation.

Patients with diabetic foot received debridement and treatments and were followed up for 24 weeks to evaluate wound healing. The following endpoints could be distinguished as rapidly healing (RH: the healing process was successfully completed within 6 months) or nonhealing (NH: the ulcer persisted or was even enlarged, development of new ulcers, amputations, or death) [5]. The group of burn victims otherwise healthy was the control which belongs to the group of RH.

For definition of the discovery cohort, we selected 12 patients with DFU who could be distinguished as RH or NH, respectively. Each NH patient was matched with one RH patient. RH patients were appropriately matched for sex, age (±5 years), diabetes duration (±5 years), and wound area (±2 cm^2^) to the studied group of NH patients in the initial discovery study. The remaining 85 patients were defined as the validation cohort 1 and were divided into two groups: NH patients (*n* = 36) and RH patients (*n* = 49), and the group of burn victims otherwise healthy was defined as the validation cohort 2.

### 2.2. Wound Exudate, Plasma, and Information Collection

Wound exudate was obtained from patients or burn victims as previously described [13]. Plasma samples from patients were collected at the first clinic visit. Wound exudate and plasma samples were stored at −20°C. We collected data on sex, age, diabetes duration, wound duration, ulcer area, body mass index (BMI), blood pressure, blood glucose, glycosylated hemoglobin (HbA1c), and lipoprotein lipid levels. Hypertension and hyperlipidemia were defined as previously described [9].

### 2.3. Targeted Protein Array

Wound exudate or plasma samples were obtained from the RH group and the NH group matched for age, sex, diabetes duration, and wound area. High-throughput protein arrays were performed on total protein (wound exudate or plasma samples) pooled from 12 RH patients or 12 NH patients, respectively. 80 different human proteins were analyzed using the RayBio Human Cytokine Antibody Array G-Series 5 (cat. no. AAH-CYT-G5-8) glass slide arrays (RayBiotech Inc., Norcross, GA, USA). An InnoScan 300 Microarray Scanner was used to scan the slides (Innopsys, Carbonne, France). Q-Analyzer software was used to process the raw fluorescence data (RayBiotech Inc., Norcross, GA, USA), using the fluorescence intensities of background and positive controls to normalize individual spot fluorescence data [9].

### 2.4. ELISA

Candidate markers were confirmed using commercial enzyme-linked immunosorbent assay (ELISA) kits (RayBiotech Inc., Norcross, GA, USA). The optical density of each well was measured on a microplate reader at 450 nm. Each wound exudate or plasma sample was diluted 1 : 2–1 : 100 into sample diluents, and duplicate assays were performed.

### 2.5. Statistics

Data were expressed as means ± SEM, median (interquartile range), or percentages (%). Categorical variables were compared using Fisher's exact test or *χ*^2^ test. Normality of the variables was compared using an independent *t*-test or Fisher-Pitman permutation test, while nonnormal distribution data were compared using a nonparametric Mann-Whitney *U* test or exact Mann-Whitney rank sum test. Correlation analysis was performed using the Pearson correlation coefficient (normally distributed variables) or Spearman correlation analysis (nonnormally distributed variables). Binary logistic regression analysis was used to assess independent predictors of wound healing. A receiver operating characteristic (ROC) curve was constructed to find the cut-off point of ENA-78 for predicting wound healing. *P* values < 0.05 were considered statistically significant. SPSS 19.0 (SPSS Inc., Chicago, IL, USA) was used to process the data.

## 3. Results

### 3.1. Patient Characteristics

All the participants were followed up for 24 weeks. 30 burn victims otherwise healthy were the control as part of the RH groups. Among the 109 patients with diabetic foot, 61 patients were defined as RH, while 48 patients were defined as NH. The main characteristics of the three groups are shown in Table 1. The initial biomarker screen of wound exudate or plasma from patients with diabetic foot (RH and NH, *n* = 12/group) was performed using protein arrays, and there were no differences in age, sex, duration of diabetes, BMI, fasting plasma glucose (FPG), ulcer area, HbA1C, diabetes duration, the prevalence of hypertension, hyperlipidemia, or wound infection between the RH and NH groups (all *P* > 0.05). In the validation cohort 1, samples from the RH group (*n* = 49) and the NH group (*n* = 36) were analyzed by ELISA, and there were significant differences between the groups in FPG and HbA1C (all *P* < 0.05) but no differences in the others (all *P* > 0.05). In the validation cohort 2, samples defined as the NH group (*n* = 30) were also analyzed by ELISA, and the ulcer area was larger than that of the patients with diabetic foot but no differences in sex or BMI. The age and FPG were significantly lower than NH of validation cohort 1.

### 3.2. High-Throughput Protein Array Analysis

**Figure 1:**
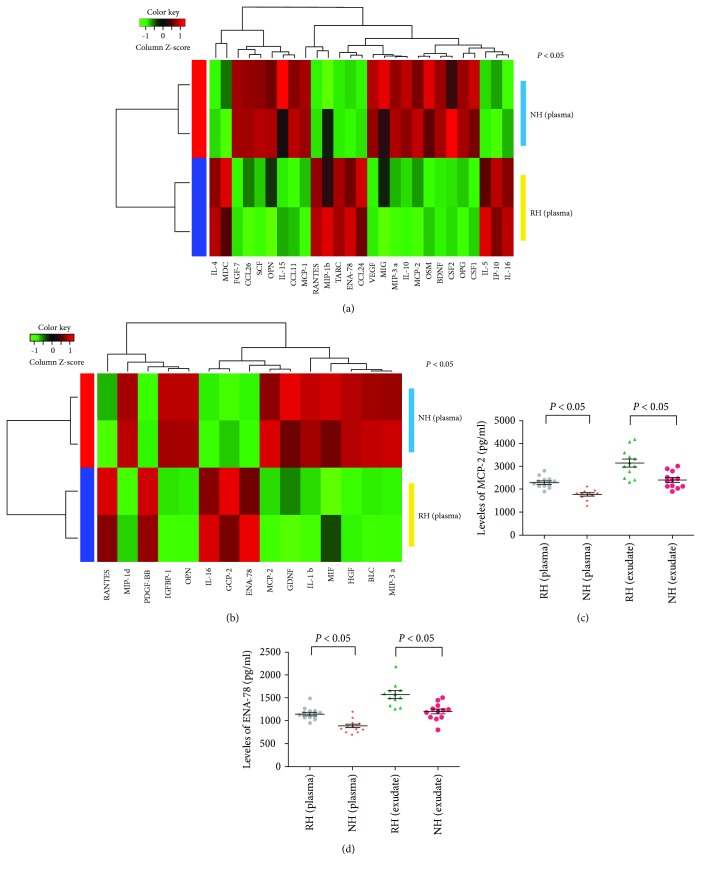
Plasma or exudate-derived protein arrays and validation experiments. (a) Heatmap of deregulated proteins in plasma pools from RH patients (*n* = 12) vs NH patients (*n* = 12). Each column under different subgroups represents a technical replicate. (b) Heatmap of deregulated proteins in exudate pools from RH patients (*n* = 12) vs NH patients (*n* = 12). Each column under different subgroups represents a technical replicate. (c, d) Validation of selected proteins in individual wound exudate or plasma sample from the 12 RH patients and 12 NH patients by ELISA, *P* < 0.05.

Using the standard 1.5-fold threshold for upregulation and the 0.6-fold threshold for downregulation, each protein was performed in duplicate assays in the slides. Levels of 27 proteins were significantly altered in protein profiling of pooled plasma of 12 RH patients versus 12 NH patients (Figure 1(a)), and levels of 15 proteins were significantly altered in protein profiling of pooled wound exudate of 12 RH patients versus 12 NH patients (Figure 1(b)). Based on the level of regulation and their reported functional roles, 2 proteins (MCP-2 and ENA-78) with the consistent trend of the change in the pooled wound exudate and the pooled plasma were selected for further validation.

### 3.3. Validation Study of Biomarkers for Wound Healing

**Figure 2:**
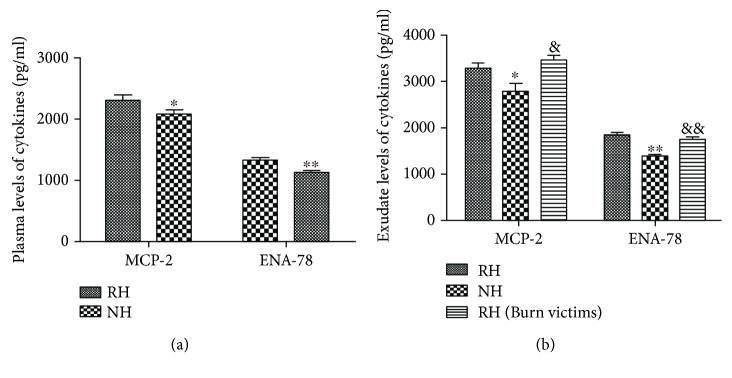
A validation study for the findings of antibody array analysis in independent cohort subjects with RH (*n* = 49) and NH (*n* = 36) patients by ELISA. MCP-2 and ENA-78 were significantly decreased in the NH group compared with the RH group. ^#^*P* < 0.05 or ^##^*P* < 0.001 versus RH. ^&^*P* < 0.05 or ^&&^*P* < 0.001 versus NH (exudate).

Levels of 2 proteins were further validated in individual wound exudate or plasma sample from the 12 RH patients and 12 NH patients by ELISA. ELISA analysis results of the 2 proteins tested in protein arrays were consistent with those of the protein arrays (including wound exudate and plasma samples) (Figures 1(c) and 1(d)). We then performed a validation study in an independent cohort of RH patients (*n* = 49), NH patients (*n* = 36), and burn victims otherwise healthy (defined as the NH group, *n* = 30) by ELISA. As shown in Table 2 and Figure 2, MCP-2 and ENA-78 were significantly decreased in the NH group compared with the RH group.

### 3.4. Analysis of Plasma or Wound Exudate ENA-78 Level and Wound Healing Risk Factors

There were negative associations between HbA1C and MCP-2 or ENA-78 (plasma or exudate, all *P* < 0.05). There were also negative associations between BMI, hypertension, or hyperlipidemia and levels of MCP-2 or ENA-78 (plasma or exudate) but did not reach the level of statistical significance (all *P* > 0.05) (Table 3).

### 3.5. Risk Factors for Wound Healing

Taking healing as the dependent variable, the traditional risk factors (sex, age, BMI, FPG, ulcer area, HbA1C, diabetes duration, hyperlipidemia, and antibiotic therapy) were entered into binary logistic regression analysis.

After adjusting for the traditional confounding risk factors, wound exudate level of ENA-78 remained having a significant association with an increased odds ratio (OR) for healing (*P* < 0.05), whereas plasma levels of ENA-78 and MCP-2 (plasma and exudate) did not reach the level of statistical significance in this study cohort (Table 4).

### 3.6. Diagnostic Value of ENA-78 for Wound Healing

**Figure 3:**
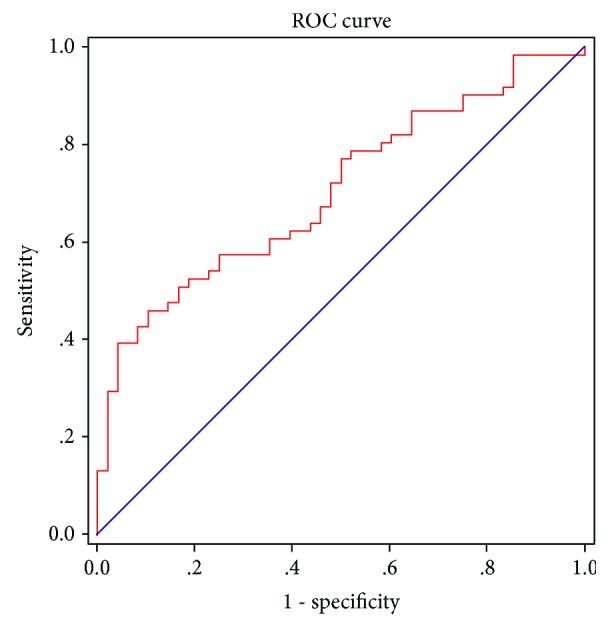
ENA-78 is a predictive factor for healing. All study subjects were included in the analysis. The AUC of ENA-78 was 0.705 (*P* < 0.001) and the optimal cut-off point for ENA-78 was 1792.00 ng/mL, with a sensitivity of 45.90% and a specificity of 89.58%.

Receiver operating characteristic (ROC) curve analysis was performed to verify the diagnostic accuracy of ENA-78 for wound healing. The area under the curve (AUC) of ENA-78 was 0.705 (95% confidence intervals (CI) 0.608–0.801, *P* < 0.001) and the optimal cut-off point for ENA-78 was 1792.00 ng/mL, which could be used as a diagnostic cut-off point in wound exudate for wound healing. At this level, the Youden index = 0.355, sensitivity was 45.90% (95% CI 0.331–0.592), and specificity was 89.58% (95% CI 0.773–0.965) (Figure 3).

## 4. Discussion

In the present study, even after adjusting for traditional confounding risk factors (sex, age, BMI, FPG, ulcer area, HbA1C, diabetes duration, hyperlipidemia, and antibiotic therapy), exudate level of ENA-78 was found to be significantly decreased in the NH group compared with the RH group. ENA-78 was independently and strongly associated with diabetic foot healing. Therefore, our findings implicate exudate level of ENA-78 as an early predictor of wound healing in patients with DFU.

Based on the patient characteristics, FPG and HbA1C were found to be significantly increased in the NH group compared with the RH group in the validation cohort 1 group, which means that glycemic control plays an important role in wound healing. Spearman's rho correlation analysis showed that there were negative associations between HbA1C and MCP-2 or ENA-78 (plasma or exudate); with the increase of HbA1C, the levels of MCP-2 or ENA-78 decreased, and the healing of the wound is becoming more difficult. After adjusting for traditional confounding risk factors, this analysis revealed that the decreased wound exudate ENA-78 was independently and strongly associated with wound healing of patients with diabetic foot. Finally, ROC curve analysis showed that the AUC of ENA-78 was 0.705, which provided further evidence confirming that ENA-78 plays an important role in the prediction model for wound healing of DFU. In our study, we also selected 30 burn victims otherwise healthy as controls; the exudate level of ENA-78 in burn victims was also higher than that in NH patients with diabetic foot, which further revealed that decreased wound exudate ENA-78 delayed the healing of the wound.

Epithelial neutrophil activator-78 (ENA-78) encoded by *CXCL5* has been shown to be chemotactic for neutrophils and stimulates neutrophilic degranulation causing the release of myeloperoxidase and generating reactive oxygen species, a key leukocytic chemokine that is both a neutrophil attractor and activator [14]. ENA-78 is involved in platelet-dependent activation of monocytes, displays angiogenic properties, and has been implicated in many diseases (obesity, diabetes, diabetic retinopathy, subclinical atherosclerosis, acute coronary syndromes, ischemic stroke, abdominal aortic aneurysm, and thrombosis) [15–19]. In this study, we identified a significant association between ENA-78 and wound healing in patients with diabetic foot even after adjustment for traditional confounding risk factors. This is the first study to demonstrate a strong relationship between the wound exudate level of ENA-78 and healing in patients with diabetic foot.

Wound healing is a complex process comprising hemostasis, inflammation, proliferation, and remodeling [20]. Different cell types, complex signaling events, and numerous growth factors are involved in each stage of wound healing [21]. Mehanni et al. reported that the rate of reepithelialization of the irradiated wounds was affected by the increased CXCL-5 expression [22]. Moreover, Mishra et al. previously reported that there was a significantly increased levels of CXCL5 and SDF-1 in the healing wounds treated with human mesenchymal stem cells as compared to normal control [23], and the interaction of keratinocytes and mesenchymal stem cells results in increased expression of CXCL5 in the healing wound [24]; however, the level of ENA-78 was significantly decreased in NH patients in our study which was consistent with the results of the above research. Several studies also reported that ENA-78 is involved in the inflammatory phase of wound healing [25–27]. Based on previous reports, CXCL5 is involved in angiogenesis [28], and the lack of ENA-78 affects angiogenesis; however, the precise molecular mechanism underlying the influence of ENA-78 on the development of wound healing remains to be determined in further prospective studies.

There are several limitations of the present study that should be considered. Firstly, although this study involved 80 potential biomarker proteins (including chemokines, inflammatory cytokines, and angiogenesis-related factors), this panel is not exhaustive. It is estimated that there are more than 10,000 proteins in the human plasma or exudate; thus, further studies are required to investigate a more comprehensive panel of plasma or exudate proteins. Secondly, although this cross-sectional study revealed that ENA-78 is associated with wound healing of patients with diabetic foot, the physiological function of ENA-78 in wound healing and its pathogenic role in nonhealing DFU remain to be determined in further prospective studies.

**Figure 4:**
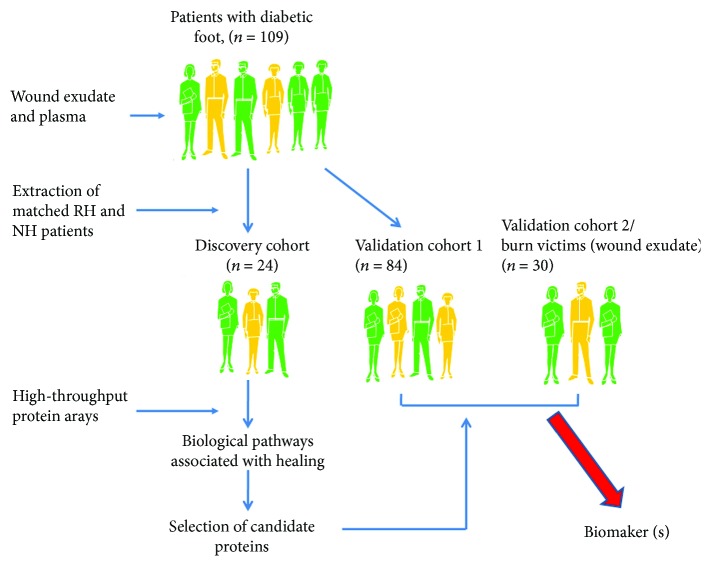
Summary of the study design. Identification and validation of candidate pathogenic factors.

In conclusion, our data show that decreased exudate ENA-78 is independently associated with wound healing of patients with diabetic foot which delays diabetic wound healing. Exudate ENA-78 level is implicated as an early predictor of wound healing of patients with diabetic foot with the potential for development of strategies for the prevention or treatment of diabetic foot (Figure 4). Further investigations are needed to exploit the physiological function of ENA-78 in wound healing and its pathogenic role in nonhealing DFU.

## Figures and Tables

**Table 1:** Clinical characteristics of the participants under investigation.

Characteristics	Discovery cohort			Validation cohort 1			Validation cohort 2	
	RH	NH	*P*	RH	NH	*P*	RH	*P*
*n*	12	12		49	36		30	
Sex, women (%)	50	58.3	0.256‡	44.9	50	0.479‡	43.3	0.589‡
Age (years)	61.42 (50.50–72.25)	58.00 ± 2.77	0.446^†^	62.78 ± 1.59	67.58 ± 1.60	*0.040*	30.10 (11.25–55.25)	*0.000 * ^†^
BMI (kg/m^2^)	24.74 ± 0.60	24.90 ± 0.82	0.877	24.50 ± 0.42	25.28 ± 0.48	0.224	24.51 ± 0.43	0.241
FPG (mmol/L)	10.27 (6.52–14.22)	9.99 (6.08–10.32)	0.917^†^	11.09 (6.73–16.49)	14.38 (10.51–17.33)	*0.012 * ^†^	4.90 ± 0.11	*0.000 * ^†^
Ulcer area (cm^2^)	3.03 (1.23–3.10)	2.78 (1.85–3.10)	0.788^†^	2.91 (1.60–2.55)	2.77 (1.30–2.60)	0.820^†^	8.21 ± 0.48	*0.000 * ^†^
HbA1C (%)	9.97 ± 0.70	8.78 (6.63–12.03)	0.313^†^	7.67 ± 0.16	10.27 ± 0.34	*0.000*	/	/
Diabetes duration (years)	8.00 ± 1.11	7.33 ± 0.78	0.629	8.54 (2.75–12.00)	8.31 (4.63–11.38)	0.869^†^	/	/
Hypertension (%)	50	50	1.000‡	32	18	0.157‡	/	/
Hyperlipidemia (%)	16.7	8.3	0.054‡	11	7	0.738‡	/	/
Antibiotic therapy (%)	41.7	41.7	1.000‡	18	19	0.140‡	/	/

FPG: fasting plasma glucose; HbA1c: glycosylated hemoglobin. Data are shown as mean ± SEM, median (interquartile range), or percentage (%) of subjects in each group. Significant values are marked in italic. Differences between the groups (RH *vs* NH) were analyzed using ^†^Mann-Whitney *U* test or ^‡^*χ*^2^ test; all the others were analyzed using *t*-tests. In validation cohort 2 group, *P* was compared with NH (validation cohort 1).

**Table 2:** Validation assays of proteins in diabetic foot or in burn victims and their potential to discriminate NH from RH (pg/mL).

Proteins	Patients with diabetic foot				Burn victims
	Plasma		Exudate		Exudate
	RH (*n* = 49)	NH (*n* = 36)	RH (*n* = 49)	NH (*n* = 36)	RH (*n* = 30)
MCP-2	2308.95 (1767.06–2263.37)	2083.21 ± 70.22^†#^	3192.30 (2548.00–3776.81)	2789.33 ± 165.25^†#^	3457.49 ± 99.84^&^
ENA-78	1331.00 ± 35.67	1134.42 (1077.42–1230.90)^†##^	1841.49 ± 54.93	1390.43 ± 26.14^##^	1755.36 (1555.67–1925.87)^†&&^

Data are shown as mean ± SEM, median (interquartile range) of subjects in each group. Differences between the groups were analyzed using ^†^Mann-Whitney *U* test; the others were analyzed using *t*-tests. ^#^*P* < 0.05 or ^##^*P* < 0.001 versus RH. ^&^*P* < 0.05 or ^&&^*P* < 0.001 versus NH (exudate).

**Table 3:** Spearman's rho correlation analysis of plasma or wound exudate cytokine levels and wound healing risk factors.

Variable		MCP-2		ENA-78	
		Plasma	Exudate	Plasma	Exudate
Age	*Rho*	−0.081	0.048	−0.042	−0.161
	*P*	0.405	0.622	0.666	0.095
Gender	*Rho*	−0.149	−0.129	−0.012	0.009
	*P*	0.121	0.181	0.899	0.928
BMI	*Rho*	−0.049	−0.100	−0.009	−0.055
	*P*	0.612	0.302	0.924	0.573
FPG	*Rho*	0.014	−0.165	−0.051	0.019
	*P*	0.881	0.087	0.597	0.846
Ulcer area	*Rho*	0.070	0.003	0.027	0.070
	*P*	0.466	0.978	0.780	0.467
HbA1C	*Rho*	−0.284	−0.275	−0.206	−0.266
	*P*	*0.003*	*0.004*	*0.031*	*0.005*
Diabetes duration	*Rho*	0.034	0.133	0.133	0.011
	*P*	0.723	0.169	0.168	0.907
Hypertension	*Rho*	−0.114	−0.026	−0.160	−0.058
	*P*	0.239	0.784	0.097	0.551
Hyperlipidemia	*Rho*	−0.107	−0.013	−0.060	−0.039
	*P*	0.267	0.891	0.534	0.686
Antibiotic therapy	*Rho*	−0.022	0.117	0.104	0.091
	*P*	0.822	0.227	0.284	0.345

All study subjects were included in the analysis. Significant values are marked in italic.

**Table 4:** Risk factors for wound healing by binary logistic regression analysis.

	OR	95% CI for OR	*P*	OR^∗^	95% CI for OR^∗^	*P* ^∗^
Sex	0.951	0.446–2.026	0.896	∕	∕	∕
Age	1.024	0.987–1.061	0.203	∕	∕	∕
BMI	1.076	0.938–1.234	0.295	∕	∕	∕
FPG	1.066	1.000–1.138	0.051	∕	∕	∕
Ulcer area	0.993	0.865–1.140	0.922	∕	∕	∕
HbA1C	1.958	1.463–2.621	*0.000*	∕	∕	∕
Diabetes duration	0.965	0.903–1.031	0.290	∕	∕	∕
Hypertension	1.652	0.767–3.557	0.199	∕	∕	∕
Hyperlipidemia	1.061	0.406–2.775	0.904	∕	∕	∕
Antibiotic therapy	0.605	0.281–1.303	0.199	∕	∕	∕
MCP-2 (plasma)	0.999	0.998–1.000	*0.003*	0.999	0.998–1.000	0.079
MCP-2 (exudate)	0.999	0.999–1.000	*0.003*	0.999	0.999–1.000	0.065
ENA-78 (plasma)	0.998	0.996–0.999	*0.013*	0.998	0.996–1.001	0.177
ENA-78 (exudate)	0.997	0.996–0.999	*0.000*	0.998	0.996–1.000	*0.018*

CI: confidence interval. Logistic regression models were used to calculate OR. ^∗^Adjusted for sex, age, BMI, FPG, ulcer area, HbA1C, diabetes duration, hyperlipidemia, and antibiotic therapy. All study subjects were included in the analysis. Significant values are marked in italic.

## Data Availability

The data used to support the findings of this study are available from the corresponding author upon request.
